# Urogenital fistulae: A prospective study of 50 cases at a tertiary care hospital

**DOI:** 10.4103/0974-7796.65114

**Published:** 2010

**Authors:** Rajkumar Mathur, Nitin Joshi, Gaurav Aggarwal, Ramsharan Raikwar, Vaibhav Shrivastava, Poonam Mathur, Poonam Raikwar, Rupali Joshi

**Affiliations:** Department of Surgery, M.G.M Medical College and M.Y.H Group of Hospitals, Indore, Madhya Pradesh - 452 001, India; 1 Department of Obstetrics and Gynecology, M.G.M Medical College and M.Y.H Group of Hospitals, Indore, Madhya Pradesh - 452 001, India; 2 ESI Hospital, Indore, Madhya Pradesh - 452 001, India

**Keywords:** Obstructed labor, urogenital fistulae, vesico vaginal fistula

## Abstract

**Introduction:**

The misfortunate incident of formation of a urogenital fistula remains a major challenge for surgical urologists worldwide. Such fistulae may not be a life-threatening problem, but surely the women face demoralization, social boycott and even divorce and separation. The fistula may be vaginal, recto-vaginal or a combination of the two. The World Health Organization (WHO) has estimated that in the developing nations, nearly 5 million women annually suffer severe morbidity with obstetric fistulae being the foremost on the list. The objective of our study was to enunciate the patient demography, patient profile, incidence, type of surgery, as well as the long-term outcomes encountered in the management of all types of genital fistulae at a tertiary care centre.

**Materials and Methods:**

50 consecutive patients, attending the outpatient department with urogenital fistulae, were studied during the period of 5 years from July 2005 to July 2009. All female patients with complaints of urinary incontinence and fecal incontinence and dribbling, patients having a history of obstructed labor, radiotherapy, instrumental delivery, foreign body or trauma and with a history of hysterectomy (abdominal/ vaginal) and lower segment caesarean section (LSCS) were included. A thorough urological examination included a dye study using methylene blue, Renal function tests, X-ray KUB and intravenous urography (IVU). Cystoscopy along with examination under anaesthesia (EUA) were done to assess the actual extent of injury. All patients were subjected to appropriate surgical interventions via the same combination of surgeons . Post operatively, prophylactic antibiotics were administered to all patients and patients were managed till discharge and followed thereafter via regular outpatient visits for a period of 3 years.

**Results:**

Age of patients ranged from 21 to 40 years. 64% patients hailed from rural areas, 76% were from the lower socio-economic strata, 40% illiterate and 69% were short Statured. Vesico vaginal fistulae (VVF) was seen in 64% cases of which 50% were due to obstructed labor, 19% cases post LSCS and 31% cases post total abdominal hysterectomy (TAH). 68% of urogenital fistulae were between 1 to 3 cms. We obtained a 75% cure rate in UVF, 87.5% cure rate in RVF while a 93.75% cure rate was observed in patients with VVF. 76% of all patients were cured while 8% had a recurrence, probably due to the large size of fistula.

**Conclusion:**

Genital fistula is preventable, yet it remains a significant cause of morbidity among females of reproductive age group. Despite facilities available, certain conditions like physical, social, economic, illiteracy, and a very casual attitude towards maternal health and children birth practices limit utilization of services for women. It is important that the modern health care providers should be aware of these aspects, so that they can recognize services that are appropriate and acceptable to the people. Thus, one must agree that in cases of urogenital fistulae, "prevention is better than cure".

## INTRODUCTION

The misfortunate incident of formation of a urogenital fistula remains a major challenge for surgical urologists worldwide. Such fistulae may not be a life-threatening problem, but surely the women face demoralization, social boycott and even divorce and separation. The fistula may be vaginal, recto-vaginal or a combination of the two.[1] The etiology of the condition has changed over the years and in developed countries obstetrical fistulae are rare and are usually a result of gynecological surgeries or radiotherapy.[2] Obstetric vesico vaginal fistulae (VVF) due to obstructed labor has long been eradicated from the developed society, but still remains a significant problem among the developing nations.[3]

The WHO has estimated that in the developing nations, nearly 5 million women annually suffer severe morbidity with obstetric fistulae being the foremost on the list.

The objective of our study was to enunciate the patient demography, patient profile, incidence, type of surgery, as well as the long term outcomes encountered in the management of all types of genital fistulae at a tertiary care centre.

## MATERIALS AND METHODS

The present study was carried out as a prospective study of urogenital fistula at the department of surgery in collaboration with the department of gynecology, M.Y. Hospital, Indore, India, during five-year period from July 2005 to July 2009, conducted on 50 consecutive patients attending the outpatient department.

This study included all female patients with complaints of urinary incontinence and fecal incontinence and dribbling. Our inclusion criteria were patients having a history of obstructed labor, radiotherapy, instrumental delivery, foreign body or trauma and with a history of hysterectomy (abdominal/vaginal) and lower segment caesarean section (LSCS). These were applicable to those patients who had already developed fistulae and were confirmed via radiological and cystoscopic examination.

Poor socioeconomic standards are those as defined as low socioeconomic standards via the modified kuppuswamy scale. Malnutrition is as implied by low body mass index (BMI).

A thorough urological examination was performed to assess the exact number, site and size of the fistula which included a dye study using methylene blue. Urine culture was carried out in each case and appropriate antibiotics administered as per the sensitivity report, when required. Routine blood investigations were done viz. hemoglobin levels and blood grouping, to rule out anemia and correction done prior to surgery as well as blood sugar levels and renal function tests. X-ray KUB was done to rule out any renal calculi and intravenous urography (IVU), to rule out renal tract anomalies.

Cystoscopy along with examination under anesthesia (EUA) was done to assess the actual extent of injury. All patients were subjected to appropriate surgical interventions via the same combination of surgeons (RKM and RSR).

Post operatively, prophylactic antibiotics were administered to all patients as per their prior sensitivity patterns or as per our institutional protocol, if cultures were sterile. All patients were managed till discharge and followed thereafter via regular outpatient visits for a period of three years.

## RESULTS

During the five-year duration, a total of 50 cases of various types of urogenital fistulae were managed via the departments of surgery and gynecology in collaboration. A majority of the patients were between 21 to 40 years of age [Table 1].

**Table 1:** Age-wise distribution of women with urogenital fistulae

Age (years)	No. of patients	Percentage
<15	2	4
20‐30	18	36
31‐40	10	20
41‐50	14	28
51‐60	6	12
Total	50	100

Demographically, 64% patients hailed from rural areas, 76% were from the lower socio-economic strata and an equal number were malnourished, 40% were illiterate and 69% were short statured, with heights less than 4' 5".

54% of females were primigravidae. The different types of fistulae encountered by us are depicted in Table 2. Out of the 50 cases of genital fistulae, vesico vaginal fistulae (VVF) was seen in 64% cases. The variety of etiologies of fistulae as seen by us is shown in Table 3. Of these, the major causes of VVF are shown in Table 4.

Out of 32 cases of VVF, 50% cases were due to obstructed labor, 19% cases post LSCS and 31% cases post total abdominal hysterectomy (TAH).

**Table 2:** Incidence of various urogenital fistulae

Type of fistula	No. of patients	Percentage
VVF	32	64
RVF	8	16
Urethrovaginal	4	8
Congenital	2	4
Vesicouterine	4	8
Total	50	100

**Table 3:** Etiological distribution of uro-genital fistulae

Cause of fistula	No. of patients	Percentage
Obstructed Labor	18	36
Post LSCS	8	16
Post TAH	14	28
Post VH	4	8
Post radiation	2	4
Others(no definable cause)	4	8
Total	50	100

**Table 4:** Causes of VVF

Cause of VVF	No. of patients	Percentage
Obstructed Labor	16	50
Post LSCS	6	19
Post TAH	10	31
Total	32	100

68% of urogenital fistulae were between 1 to 3 cms in maximum dimension. The percentage distribution of patients, in relation to their fistula size is tabulated in Table 5.

**Table 5:** Patient distribution in relation to their fistula size

Size (cm)	No. of patients	Percentage
0.5 to 1	10	20
1‐2	16	32
2‐3	18	36
3‐4	4	8
>4	2	4
Total	50	100
Total	50	100

Out of the 50 cases 28% had a single fistula while 72% had multiple fistulae. Of these, 28% cases had undergone transvaginal repair, 44% transabdominal, 16% had undergone both and 12% were treated conservatively.

Our results are depicted in Table 6, with a 75% cure rate in UVF, 87.5% cure rate in RVF while a 93.75% cure rate was observed in patients with VVF.

**Table 6:** Post management results

Fistula	No. of patients	Cured cases	Percentage
Urethrovaginal fistula	4	3	75
Rectovaginal fistula	8	7	87.5
Vesicovaginal fistula	32	30	93.75
Other	6	5	83

76% of all patients were cured while 8% had a recurrence, probably due to the large size of fistula. Patient attrition during follow up is well-known and we were also not exempt, with 10% attrition, as we lost 5 patients in follow-up.

## DISCUSSION

A vast majority of cases of urogenital fistulae occurring mainly in the developing countries, result from obstetrical causes.

On account of poverty, illiteracy and to some extent ignorance among the people in developing nations, a large number of the total births are still conducted by dai's or traditional birth attendants.

Birth trauma occurs due to prolonged and difficult home deliveries that can cause ischemic necrosis of the vaginal vault and the posterior bladder wall, causing them to slough off and passage of urine per vaginum.

Among the developed nations, with advanced health care facilities, most fistulae are the result of gynecological procedures while a few may occur following cervical cancer and radiotherapy for it. In Asia majority fell within 20-24 year age group (WHO1991).

A study from Africa in 1992 reported the occurrence of 93% of fistulae in young women and primiparas,[4] correlating well with our study. A Nigerian study[5] reported 96.5% of urogenital fistulae were associated with labor and delivery, with most patients being thin, short statured, illiterate, malnourished, poor, from a rural region and mostly developed fistulae as a primigravida during labor.[5] The single most important economic factor contributing to the prevalence of VVF is poverty, especially poverty in rural areas. According to the WHO 1991 Report on Obstetric Fistulae, women with fistulae come almost exclusively from poor families and communities.

Malnutrition attributes greatly to cause genital fistulae, as supported by evidence found in the 1981 Murphy and Baba Thakur study.

With increased literacy and improved surgical expertise, there is an increasing tendency for early repair of fistulae[6] and subsequently decreased complications and improved social life.

Urogenital fistulae are of varied types, with VVF being the most common. On the suspicion of a VVF, a thorough vaginal examination should be done, in order to identify the exact size and location, especially in relation to the trigone and also to eliminate an ureterovaginal fistula, which can be seen in as much as 10% cases as an association.

There are varied approaches for repair of these urogenital fistulae viz. abdominal and vaginal. The abdominal approach may be used to treat all types of VVF and preferred when concomitant ureteric reimplantation is necessary. The vaginal approach involves a tension free fistula closure with or without tract excision, forming an anterior vaginal wall flap and use vascularized grafts interposed in between, when required.[7,8]

Fistula size also plays an equally important role in wound healing, wherein small fistulae may heal on conservative measures as retaining foleys catheters and head end elevation of the bed.[9]

## CONCLUSION

Genital fistula is preventable, yet it remains a significant cause of morbidity among females of reproductive age group. Despite facilities available, certain conditions like physical, social, economic, illiteracy, and a very casual attitude towards maternal health and children birth practices, limit utilization of services for women.

The poor socio-economic development is the basic underlying factor responsible for maternal ill health and genital fistulae. Standards of health in developing countries are low which is compounded by malnutrition and infections, particularly in isolated rural areas where there is unequal distribution of government sources and lack of basic infrastructure.

Curative health does not eradicate the problem, it only soothes it. Preventive health care encourages participation of potential users of health care as it involves intensive health education of the community. It involves health care representative discuss the problems in their communities with the people and suggesting how they can participate in solving them. The establishment of hospitals in rural centers encourages better accessibility of genital fistula patients, as under that environment the associated social stigma can be easily dealt with. In most societies, cultural and spiritual aspects of pregnancy and childbirth have a strong influence on behavior.

It is important that the modern health care providers should be aware of these aspects, so that they can recognize services that are appropriate and acceptable to the people.

Traditional birth attendants should also be trained to recognize and refer obstetric complications without undue delay. Thus, one must agree that in cases of urogenital fistulae, "prevention is better than cure".
